# Epidemiology of maxillofacial trauma in elderly patients receiving oral anticoagulant or antithrombotic medication; a Swiss retrospective study

**DOI:** 10.1186/s12873-024-01039-1

**Published:** 2024-07-18

**Authors:** David Bettschen, Dimitra Tsichlaki, Eleftherios Chatzimichail, Jolanta Klukowska-Rötzler, Martin Müller, Thomas C. Sauter, Aristomenis K. Exadaktylos, Mairi Ziaka, Michael Doulberis, John-Patrik Burkhard

**Affiliations:** 1 Department of Emergency Medicine, Inselspital, University Hospital Bern, University of Bern, 3010 Bern, Switzerland; 2 Department of Ophthalmology, University Hospital of Basel, Basel, Switzerland; 3 Gastroklinik, Private Gastroenterolgy Practice, 8810 Horgen, Switzerland; 4 Division of Gastroenterology and Hepatology, Medical University Department, Kantonsspital Aarau, 5001 Aarau, Switzerland; 5 Limat Cleft and Craniofacial Centere, 8005 Zurich, Switzerland

**Keywords:** Geriatric patients, Elderly falls, Anticoagulation, Antiplatelet therapy, Maxillofacial injury, Bleeding complications, Fractures

## Abstract

**Background:**

The percentage of elderly trauma patients under anticoagulation and antiplatelet agents has been rising lately. As newer agents are introduced, each comes with its own advantages and precautions. Our study covered elderly patients admitted to the ED with maxillofacial trauma while on anticoagulation (AC) or antiplatelet therapy (APT). We aimed to investigate the demographic characteristics, causes, and types of maxillofacial trauma, along with concomitant injuries, duration of hospitalisation, haemorrhagic complications, and the overall costs of care in the emergency department (ED).

**Methods:**

Data were gathered from the ED of Bern University Hospital. In this retrospective analysis, patients over 65 of age were included, who presented at our ED with maxillofacial trauma between 2013 and 2019 while undergoing treatment with therapeutic AC/APT.

**Results:**

The study involved 188 patients with a median age of 81 years (IQR: 81 [74; 87]), of whom 55.3% (*n*=104) were male. More than half (54.8%, *n*=103) were aged 80 years or older. Cardiovascular diseases were present in 69.7% (*n*=131) of the patients, with the most common indications for AC/APT use being previous thromboembolic events (41.5%, *n*=78) and atrial fibrillation (25.5%, *n*=48). The predominant cause of facial injury was falls, accounting for 83.5% (*n*=157) of cases, followed by bicycle accidents (6.9%, *n*=13) and road-traffic accidents (5.3%, *n*=10). The most common primary injuries were fractures of the orbital floor and/or medial/lateral wall (60.1%, *n*=113), zygomatic bone (30.3%, *n*=57), followed by isolated orbital floor fractures (23.4%, *n*=44) and nasal bone fractures (19.1%, *n*=36). Fractures of the mandible occurred in 14.9% (*n*=28). Facial hematomas occurred in 68.6% of patients (129 cases), primarily in the midface area. Relevant facial bleeding complications were intracerebral haemorrhage being the most frequent (28.2%, *n*=53), followed by epistaxis (12.2%, *n*=23) and retrobulbar/intraorbital hematoma (9%, *n*=17). Sixteen patients (8.5%) experienced heavy bleeding that required emergency treatment. The in-hospital mortality rate was 2.1% (4 cases).

**Conclusions:**

This study indicates that falls are the leading cause of maxillofacial trauma in the elderly, with the most common diagnoses being orbital, zygomatic, and nasal fractures. Haemorrhagic complications primarily involve facial hematomas, especially in the middle third of the face, with intracerebral haemorrhage being the second most frequent. Surgical intervention for bleeding was required in 8.5% of cases. Given the aging population, it is essential to improve prevention strategies and update safety protocols, particularly for patients on anticoagulant/antiplatelet therapy (AC/APT). This can ensure rapid diagnostic imaging and prompt treatment in emergencies.

## Introduction

As the global population continues to age, it is of paramount importance to bear in mind how susceptible the elderly are to accidents and injuries [1]. Age-related physiological changes in the musculoskeletal system, sensory functions, and overall frailty heighten the risk of accidents, particularly falls, which are a predominant cause of the greater morbidity and mortality among older adults [2–4]. Indeed, more than 25% of older individuals experience fall annually, and approximately 27,000 older adults die from fall-related injuries each year [5]. The economic impact is also substantial. In the Netherlands, for example, the mean financial burden of falls in the elderly population has been estimated as approximately 674.5 million euros per year [6]. While there are many publications on fall-related injuries in the elderly, there remains a need for comprehensive understanding of the complex interplay between age-related factors, injury patterns, and potential interventions to mitigate the impact of these injuries [2, 3].

In the realm of anticoagulant and antiplatelet therapies, two distinct categories emerge: direct oral anticoagulants (DOACs) and vitamin K antagonists (VKAs), juxtaposed to antiplatelet agents such as P2Y12 inhibitors, acetylsalicylic acid (ASS), and heparin. These therapeutic classes play pivotal roles in managing various cardiovascular and thrombotic conditions, though they differ in mechanisms, administration, indications, and risk profiles [7].

Among the two available primary classes of oral anticoagulants, VKAs are the longest-standing option, having been used since the 1940s with the approval of warfarin for treating venous thromboembolism (VTE). DOACs have emerged more recently as an alternative to VKAs [8]. DOACs and VKAs predominantly address conditions like atrial fibrillation, and preventing VTE and strokes [9]. P2Y12 inhibitors and ASS are integral in managing atherothrombotic conditions, such as acute coronary syndromes and stent placement, and provide a safeguard against arterial clot formation [10]. In its various forms, heparin is still the prominent treatment for acute thromboembolic events and in perioperative settings [11, 12].

The percentage of elderly trauma patients on AC/APT has recently increased. Since 2009, there has been a rise in ED presentations involving patients on newer oral anticoagulants, such as factor Xa inhibitors. These newer agents offer simplified management and more rapid onset of the therapeutic effect [13]. However, their predisposition to bleeding, coupled with the lack of a specific antidotes or the exceptionally high price of the existing antidotes, presents significant challenges in clinical practice and raises concerns about the widespread use of these agents [14–16].

Considering the emerging prevalence of atherothrombotic disease and conditions prone to thromboembolic events as the population ages, it is crucial to enhance strategies for AC/APT and to better understand its complications in this vulnerable demographic [17]. There remains a divergence in expert opinions regarding the definitive benefits of DOACs in elderly patients. It is pertinent to note that clinical trials assessing AC therapies tend to underrepresent older adults. Nonetheless, the expanded use of newer anticoagulants among geriatric patients and their participation in recent clinical trials have broadened our understanding about the use of these pharmaceutical agents in elderly patient [18, 19].

As already mentioned above, falls are the primary cause of facial fractures in older adults , who are more likely to suffer greater morbidity from this trauma than are younger individuals [4, 20, 21], yet research on this topic remains limited. Similarly, the available scientific literature on bleeding complications following maxillofacial trauma in elderly patients on AC/APT is rather sparse.

This study aimed to retrospectively investigate the epidemiology of maxillofacial trauma in geriatric patients on AC/APT, examining the underlying mechanisms, the causes, and patterns of trauma, haemorrhagic complications, treatment and clinical outcomes. We also sought to analyse injuries in relation to demographic characteristics such as age and gender to identify further areas of research on prevention strategies and safety considerations.

## Material & methods

### Study design and setting

This retrospective cohort study was conducted at the interdisciplinary adult ED of the University Hospital in Bern (also called “Inselspital”), Switzerland, for the timeframe of 2013– 2019.

### Inclusion and exclusion criteria

Eligible participants for this study were patients aged 65 and older who presented with maxillofacial trauma to the adult ED of the University Hospital of Bern from January 1, 2013, to December 31, 2019, while undergoing AC or APT. Patients with missing data were excluded, even when they met the requirements for age or medication. Patient were also excluded if they lacked consent for use of data as part of the general consent process.

### Study outcomes

The primary outcome of the study was the classification of maxillofacial trauma, including facial fractures and associated injuries, along with relevant bleeding complications such as intracerebral haemorrhage, epistaxis, oral bleeding, retrobulbar/intraorbital and/or soft tissue hematoma. We also documented the number of emergency interventions performed for these haemorrhages.

Secondary outcomes included i) patients demographics, ii) type of AC/APT, iii) indication for AC/APT, iv) mechanism of injury, v) concomitant injury including fractures of the upper and lower extremities, chest injury and abdominal injury , vi) major symptoms associated with maxillofacial trauma, vii) procedural outcomes including length of hospital stay and hospitalisation costs; viii) clinical outcomes, such as the Facial Injury Severity Score (FISS), in-hospital-, and 30-day mortality.

### Data handling

Patients records from the ED were stored in the clinical application “E.care” for Microsoft® Windows® (E.care BVBA, ED 2.1.3.0, Turnhout, Belgium). Data from these records were extracted to an Excel® spreadsheet (Microsoft® Excel for Mac 2019, Microsoft Corporation, Redmond, WA, USA) for analysis. We conducted a comprehensive keyword search across the entire patient database covering the duration of the study. This search encompassed all generic and brand names of oral anticoagulant and antiplatelet medications approved in Switzerland. We also accounted for spelling variations and common errors, incorporating DOACs such as apixaban, dabigatran, edoxaban, and rivaroxaban; VKAs such as acenocoumarol, phenprocoumon, and warfarin; antiplatelet agents including acetylsalicylic acid, ticagrelor, and clopidogrel.

After conducting an initial search, we proceeded with a thorough keyword search within the dataset to pinpoint instances of maxillofacial trauma. Cases meeting the inclusion criteria were examined. The findings from these searches underwent independent review and validation by two investigators, adhering to predefined inclusion and exclusion criteria. In cases of discrepancy, a senior author intervened to reach consensus.

### Data extraction

Clinical and demographic data, including age, gender, nationality, date of admission, and length of hospital stay, were extracted from patient records. All datasets were anonymised prior to analysis. Diagnoses were categorised into primary injuries, including facial fractures, and secondary trauma-associated injuries, which included relevant facial bleeding complications such as epistaxis, retrobulbar/soft tissue haematoma, intracerebral hemorrhage, as well as fractures of the upper and lower extremity, chest injury, spinal and abdomen injury.

**Fig. 1:**
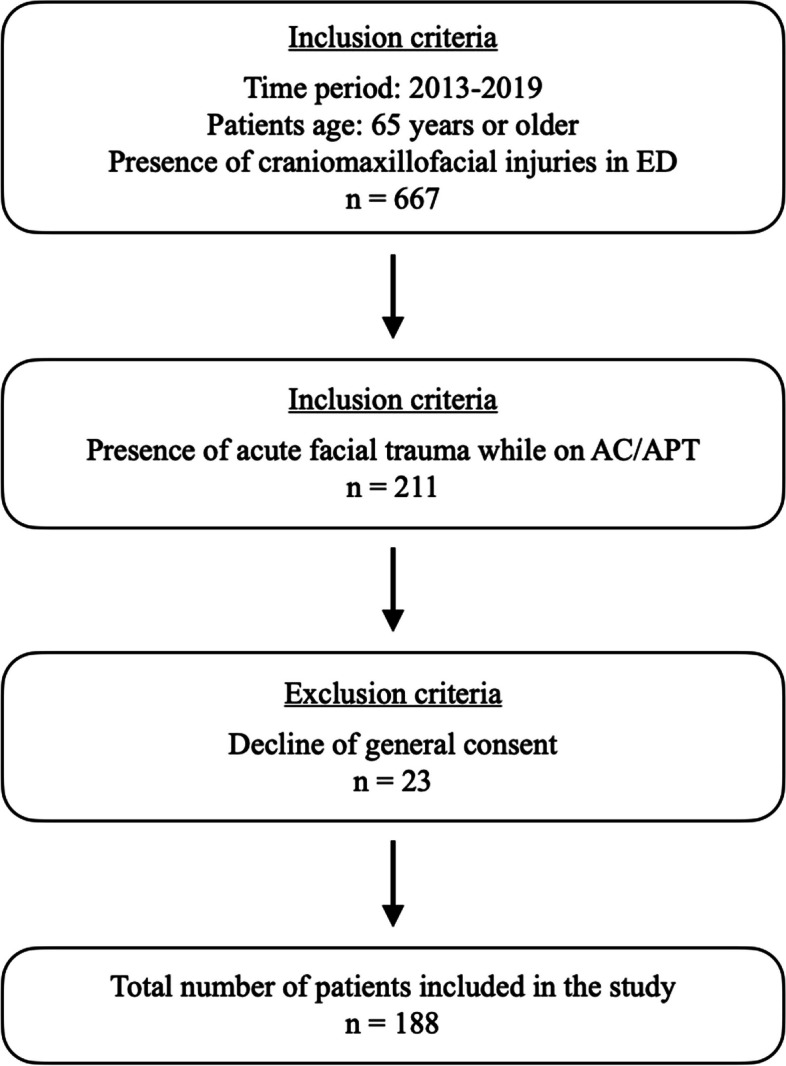
Fracture categorisation was modified based on the facial thirds: In the upper third (blue), are included the skull base, orbital roof, calvaria, and frontal sinus. The middle third (red) covers central midface fractures, such as LeFort I and II, the naso-orbito-ethmoid (NOE) complex, and nasal bone fractures; centro-lateral midface at the LeFort III level, and lateral midface fractures as the zygoma; fractures isolated to the orbit as well as dental injuries are listed separately. The lower third (green) includes fractures of the mandible. It is possible for a single patient to suffer from multiple types of fractures

Facial fractures were categorised according to a modified FISS classification, as illustrated in Fig. 1. The recorded mechanisms of injury included falls (either from a standing position or from a great height), road-traffic accidents (RTA) involving car or motorcycle incidents, being hit by a car accident, bicycle accidents; assault, sports-related injuries (including skiing or snowboarding, and horse riding accident) and suicide [21–23].

Additionally, patient evaluation covered ED diagnosis, bleeding characteristics, major symptoms, concomitant injuries, and comorbidities, indication for AC/APT, way of presentation, and medication at the time of admission. The ISS, the hospitalisation costs, and the in-hospital and 30-day mortality were also calculated for all patients.

### Statistical analysis

For the descriptive analysis, the distribution of continuous variables was described as the median and interquartile range (IQR), as these variables were not normally distributed. The distribution of categorical data was reported as numbers and percentages. Categorical variables between ordinal groups were compared using the chi-square (χ^2^) test or Fisher's exact test as appropriate. The threshold of significance was set at *p* < 0.05 (two-tailed). The Wilcoxon rank sum test was used to compare medians or continuous variables between two groups. Odds ratios (OR) for all parameters were calculated with logistic regression for binary outcomes. Multivariable associations between study outcomes (30-day mortality, in-hospital mortality) and demographic characteristics such as age and gender were analysed by logistic regression (OR as a measure of the strength of association). The statistical analysis was performed using Stata® 16.1 (StataCorp, The College Station, TX, USA).

### Ethics

The last revision of the principles of the Declaration of Helsinki and Guidelines of Good Clinical Practice was fulfilled [24, 25]. The cantonal (district) ethics committee approved the study in Cantonal Ethics Committee in Bern (number 073/2015). This was a study with retrospective design and all data were anonymized prior to analysis. Because of the use of coded routine care patient data, no informed consent is needed according to Swiss law.

## Results

**Fig. 2:**
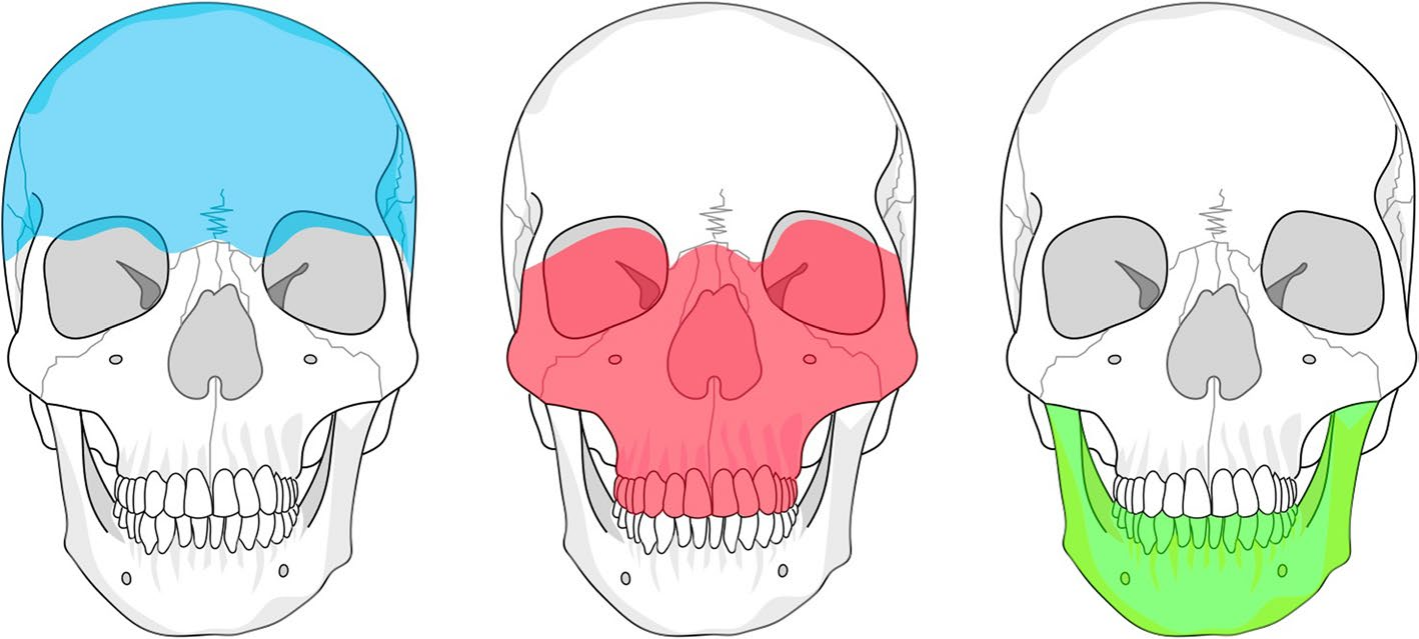
Flowchart illustrating the number (n) of patients included in this retrospective study

Between 2013 and 2019, our emergency department served 667 patients aged 65 and older for cranio-maxillofacial trauma, of whom 211 presented with acute facial trauma associated with AC/APT. Of these, 23 did not meet the inclusion criteria due to refusal of general consent and were therefore excluded from the study. A total of 188 patients were eligible and were recruited for the statistical analysis (Fig. 2).

### Demographic characteristics: age and sex distribution

In our study, 55.3% (*n*=104) of patients were male, and 44.7% (*n*=84) were female (*p*<0.001) with a male-to-female ratio of 1.24:1, as detailed in Table 1. The most frequent age group was 85-89 years, comprising 26.1% of participants (*n*=49), while 54.8% (*n*=103) were older than 80 years. The median age was 81 years (IQR: 81 [74; 87]), ranging from 65 to 96 years. Women were older than men, with a median age of 84 years (IQR: 84 [75.5; 87]) compared to the men’s median age of 79 years (IQR: 79.5 [74; 86]), *p*=0.065).

**Table 1:** Demographics including age and gender distribution

**Demographics**	**Total**		**Male**		**Female**		**p**
	***n*=188**	**[100%]**	***n*=104**	**[100%]**	***n*=84**	**[100%]**	
**Age distribution**							
65-69	23	[12.2]	16	[15.4]	7	[8.3]	
70-74	30	[16.0]	18	[17.3]	12	[14.3]	
75-79	32	[17.0]	18	[17.3]	14	[16.7]	
80-84	36	[19.1]	21	[20.2]	15	[17.9]	
85-89	49	[26.1]	22	[21.2]	27	[32.1]	
90+	18	[9.6]	9	[8.7]	9	[10.7]	0.459
**Gender**							
Male	104	[55.3]	104	[100.0]	0	[0.0]	
Female	84	[44.7]	0	[0.0]	84	[100.0]	<0.001*

### Type of referral

The majority of referrals to the ED were via ambulance (44.1%, *n*=83) or admission from other hospitals (37.8% *n*=71). Only 19 patients (10.1%) admitted themselves to the ED. Other reported referral sources included family doctor (2.7%, *n*=5), Swiss Air-Rescue (3.2%, *n*=6) or internal outpatient clinics at the University Hospital (2.1%, *n*=4). No significant differences were observed between genders (*p*=0.678).

### Time and day of consultation

Daily consultation times peaked between 06:00 to 12:00 (36.2%, *n*=68) and 12:00 to 18:00 (39.4%, *n*=74), with a lower peak between 18:00 and 00:00 (19.7%, *n*=37), none of which reached statistical significance (*p*=0.509). The frequency of events was similar from Monday to Saturday, with a decrease on Sundays. Refer to Table 2 for further details.

**Table 2:** Displays the distribution of admissions in detail across the study time period

**Admission characteristics**	**Total**		**Male**		**Female**		***p***
	***n*=188**	**[100%]**	***n*=104**	**[100%]**	***n*=84**	**[100%]**	
**Weekday**							
Monday	33	[17.6]	21	[20.2]	12	[14.3]	
Tuesday	24	[12.8]	12	[11.5]	12	[14.3]	
Wednesday	32	[17.0]	21	[20.2]	11	[13.1]	
Thursday	28	[14.9]	15	[14.4]	13	[15.5]	
Friday	27	[14.4]	13	[12.5]	14	[16.7]	
Saturday	25	[13.3]	12	[11.5]	13	[15.5]	
Sunday	19	[10.1]	10	[9.6]	9	[10.7]	0.708
**Daytime**							
00:01 – 06:00	9	[4.8]	4	[3.8]	5	[6.0]	
06:00 – 12:00	68	[36.2]	40	[38.5]	28	[33.3]	
12:01 – 18:00	74	[39.4]	43	[41.3]	31	[36.9]	
18:01 – 00:00	37	[19.7]	17	[16.3]	20	[23.8]	0.509
**Month**							
January	13	[6.9]	4	[3.8]	9	[10.7]	
February	19	[10.1]	13	[12.5]	6	[7.1]	
March	12	[6.4]	6	[5.8]	6	[7.1]	
April	15	[8.0]	8	[7.7]	7	[8.3]	
May	18	[9.6]	9	[8.7]	9	[10.7]	
June	9	[4.8]	6	[5.8]	3	[3.6]	
July	22	[11.7]	14	[13.5]	8	[9.5]	
August	20	[10.6]	10	[9.6]	10	[11.9]	
September	13	[6.9]	6	[5.8]	7	[8.3]	
October	18	[9.6]	12	[11.5]	6	[7.1]	
November	16	[8.5]	8	[7.7]	8	[9.5]	
December	13	[6.9]	8	[7.7]	5	[6.0]	0.719

### Triage

Regarding the Swiss Emergency Triage Scale [26], the majority of patients (48.9%, *n*=92) were assigned to urgent triage, with 37.2% (*n*=70) categorised as emergent. Thirteen patients (6.9%) had injuries so severe that they were triaged as acute life-threatening.

### Mechanisms of injury

Falls were the most frequent cause of injury, accounting for 157 cases (83.5%), followed by bicycle accidents (6.9%, *n*=13) and RTAs (5.3%, *n*=10). Other documented causes included horse riding incidents (1.1%, *n*=2), assaults (0.5%, *n*=1), and self-inflicted gunshot with suicidal intent (1.1%, *n*=2). When analysed by gender, bicycle accidents were significantly more prevalent among male geriatric patients (11.5%, *n*=12) compared to females (1.2%, *n*=1) suffering from maxillofacial trauma (*p*=0.001). Moreover, RTAs – including those involving motorcycles and cars - were considerably more frequent among males (7.7%, *n*=8) compared to females (2.4%, *n*=2). There were two documented cases of gunshot suicides among two male patients, while no such cases were reported among females.

### Medical history of the patients

As regards the medical history of the patients, 131 individuals (69.7%) had a cardiovascular disease. Other prevalent coexisting medical conditions included metabolic (11.2%, *n*=21), pulmonary diseases (4.3%, *n*=8), mental health conditions (3.7%, *n*=7), and neurological diseases (3.7%, *n*=7). No statistical differences were observed between genders (*p*=0.170).

### Type of blood thinners and indication

The distribution of blood thinners shows that 139 patients (73.9%) were on antiplatelet therapy (APT), with men (70.2%, *n*=73) and women (78.6%, *n*=66) receiving this medication at roughly equal rates. For oral anticoagulants (AC), fewer patients were treated, totalling 49 (26.1%), with men (29.8%, *n*=31) which is slightly more often than for women (21.4%, *n*=18). There were no significant differences between men and women (*p*=0.193). Detailed information is given in Table 3.

**Table 3:** Listing of blood thinner types

**Blood thinners type**	**Total**		**Male**		**Female**		
	***n*=188**	**[100%]**	***n*=104**	**[100%]**	***n*=84**	**[100%]**	
**NOAC**	29	[15.4]	21	[20.2]	8	[9.5]	0.044
Rivaroxaban	28	[14.9]	20	[19.2]	8	[9.5]	0.063
Apixaban	4	[2.1]	2	[1.9]	2	[2.4]	0.829
**VKA**	24	[12.8]	12	[11.5]	12	[14.3]	0.575
Phenprocoumon	22	[11.7]	10	[9.6]	12	[14.3]	0.322
Acenocoumarol	2	[1.1]	2	[1.9]	0	[0.0]	0.201
**Heparins**	0	[0.0]	0	[0.0]	0	[0.0]	-
Enoxaparin	0	[0.0]	0	[0.0]	0	[0.0]	-
Enoxaparin sodium	0	[0.0]	0	[0.0]	0	[0.0]	-
Nadroparin	0	[0.0]	0	[0.0]	0	[0.0]	-
**Clopidogrel**	17	[9.0]	12	[11.5]	5	[6.0]	0.184
**Ticagrelor**	1	[0.5]	0	[0.0]	1	[1.2]	0.265
**Acetylsalicylic acid**	124	[66.0]	63	[60.6]	61	[72.6]	0.083

Previous thromboembolic events (42.6%, *n*=80) and atrial fibrillation (25%, *n*=47) were the most reported indications for using AC/APT. Mechanical heart valve and vascular replacement were documented in only 12 patients (6.4%).

### Localisation and type of injury

The upper third included fractures of the skull base, orbital roof, frontal sinus, and calvarium. The middle third consisted of central, centro-lateral, and lateral midface fractures, with isolated orbital fractures examined independently. The lower third primarily involve mandibular fractures, whereby a distinction was made between collum and body fractures. Dental/alveolar bone injuries were also recorded separately for each jaw.

The orbit was the most frequently injured anatomical region, affecting 113 patients (60.1%); this includes cases with central, centro-lateral and lateral midface fractures that also impacted the orbit. Injuries to the orbital roof in the area of the frontal bone was significantly more affected in men (10.6%, *n*=11, *p*<0.009). Skull base fractures occurred in 17 cases (9%). Fractures of the zygomatic bone were seen in 57 patients (30.3%), followed by isolated orbital fractures in 44 patients (23.4%) and nasal bone fractures in 19.1% (*n*=36), which were the most reported primary injuries to the central midface. The mandible was affected in 28 patients (14.9%).

Associated extracranial injuries comprised upper extremities (31.4%, *n*=59), lower extremities (13.8%, *n*=26), as well as thoracic (14.4%, *n*=27), and abdominal injuries (2.1%, *n*=4). Fractures to the extremities were noted in 50 cases (26.6%) while the spinal column was affected in 5 patients (2.7%). Thoracic injuries were significantly more prevalent in men (21.2%, *n*=22, *p*=0.003).

### Bleeding complications and the need for emergency treatment

In terms of bleeding complications, intracerebral haemorrhage affected 53 patients (28.2 %), with women being significantly (*p*=0.022) more affected than men, while retrobulbar bleeding was observed in 17 patients (9%). Here as well, women were significantly more frequently affected than men (*p*=0.008). Other relevant facial bleedings such as epistaxis was encountered in 23 cases (12.2%).

**Fig. 3:**
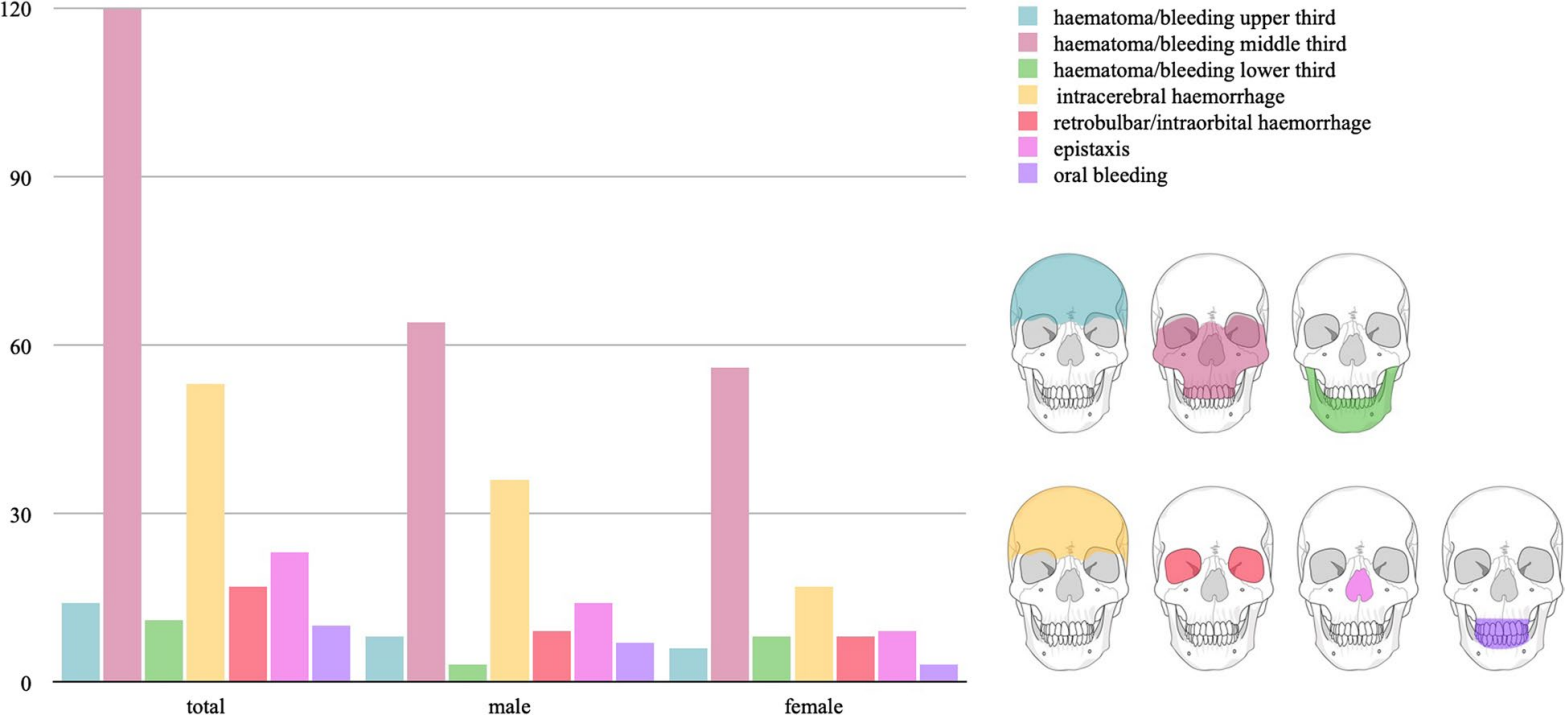
Diagram illustrating the distribution of haematomas and haemorrhages across the facial thirds

To better understand the nature of maxillofacial trauma in geriatric patients under AC/APT, we also categorised subgroups based on the affected region of the face. Haematomas primarily involved the middle third of the face in 120 patients (63.8%), followed by the lower third of face (5.9%, *n*=11). Haematomas of the upper third of face was observed in 14 patients (7.4%), see Fig. 3 or Table 4.

**Table 4:** Type and number of bleeding complications

**Bleeding complication & intervention**	**Total**		**Male**		**Female**		***p*-value**
	***n*=188**	**[100%]**	***n*=104**	**[100%]**	***n*=84**	**[100%]**	
Hematoma upper third	14	[7.4]	8	[7.7]	6	[7.1]	0.887
Hematoma middle third	120	[63.8]	64	[61.5]	56	[66.7]	0.467
Hematoma lower third	11	[5.9]	3	[2.9]	8	[9.5]	0.054
Intracranial haemorrhage	53	[28.2]	33	[23.7]	20	[40.8]	0.022
Epistaxis	23	[12.2]	14	[13.5]	9	[10.7]	0.568
Oral bleeding	10	[5.3]	7	[6.7]	3	[3.6]	0.337
Otohaematoma	2	[1.1]	2	[1.9]	0	[0.0]	0.201
Retrobulbar/intraorbital haemorrhage	17	[9.0]	8	[5.8]	9	[18.4]	0.008
**Emergency treatment for bleeding**	16	[8.5]	10	[9.6]	6	[7.1]	0.546
Orbital decompression	6	[1.6]	2	[1.9]	4	[4.8]	0.271
Cranial decompression	3	[1.6]	2	[1.9]	1	[1.2]	0.690
Epistaxis treatment	5	[2.7]	4	[3.8]	1	[1.2]	0.261
Relief of Otohaematoma	2	[1.1]	2	[1.9]	0	[0.0]	0.201
Others	1	[0.5]	1	[1.0]	0	[0.0]	0.368

Table 4 shows the distribution of patients who received bleeding-related emergency treatment, with no significant difference observed between the genders. One patient experienced a haemorrhage due to severance of a femoral artery, necessitating immediate treatment. Regarding maxillofacial injuries, 15 patients undergoing AC/APT required emergency treatment, constituting approximately 8%.

### Clinical symptoms

**Fig. 4:**
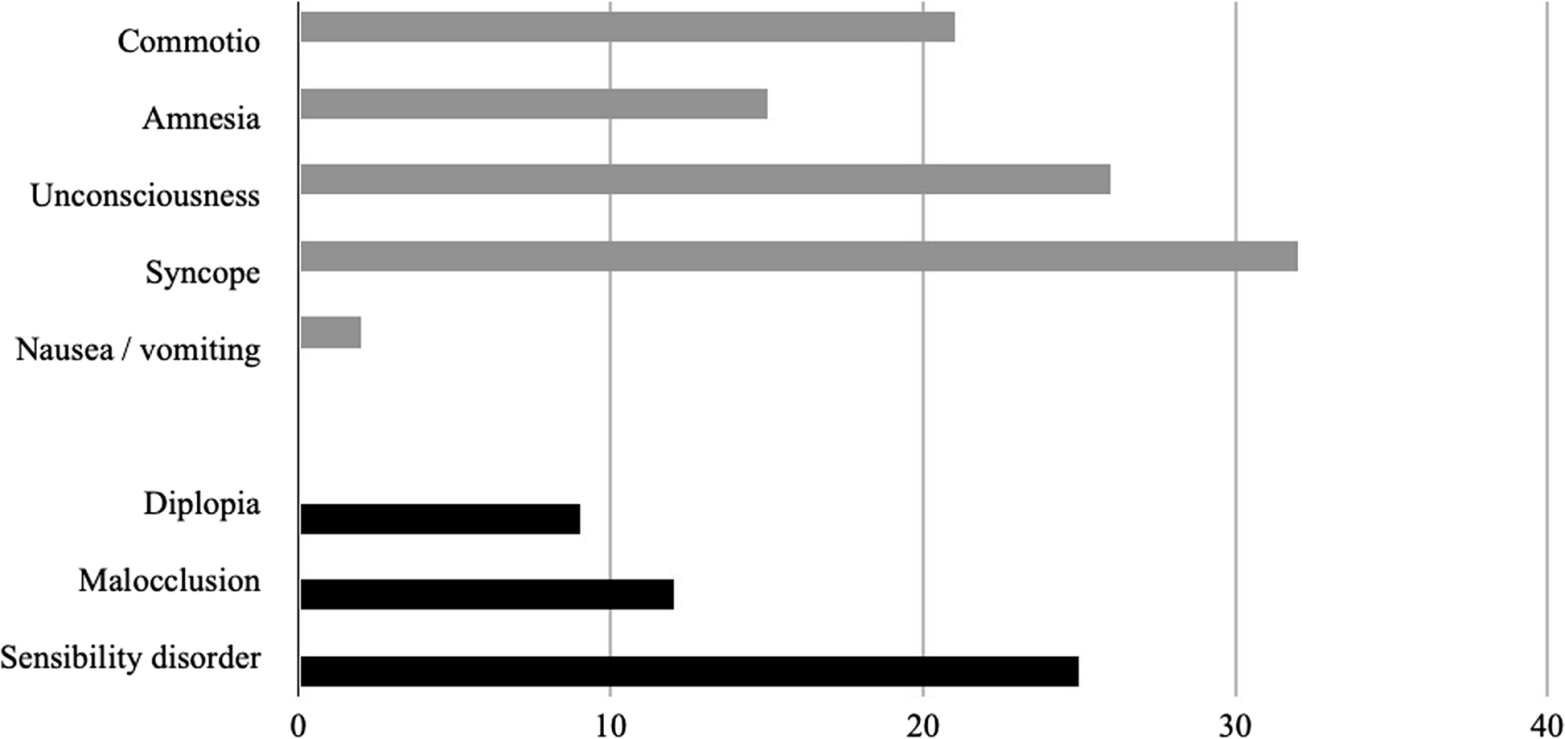
Frequently reported clinical symptoms: general symptoms are shaded in grey, and maxillofacial injury-related symptoms are shaded in black (in absolute numbers)

The most common fracture-related symptoms (Fig. 4) reported included sensibility alterations (13.3%, *n*=25), neurological disturbances such as unconsciousness (13.8%, *n*=26), and amnesia (8.0%, *n*=15), along with nausea and vomiting (*n*=2; 1.1%) indicative of commotio cerebri (11.2%, *n*=21). Sensibility alterations were significantly more prevalent in women (19%, *n*=16) than for men (8.7%, *n*=9, *p*=0.037). Out of these 25 cases involving sensibility alterations, the trigeminal nerve was affected in 23 cases, while the remaining two involved alterations in the sensitivity of the extremities. These sensibility changes were consistently documented in medical reports along with other symptoms and signs. Only one patient reported a sensory disturbance in the area of the face prior to the accident.

### Hospitalisation, mortality, ISS and FISS

Hospitalisation was necessitated for 60.6% (*n*=114) of the patients, with a gender distribution of 60.7% female (*n*=51) and 60.6% male (*n*=63). The average hospital stay was 5 days (IQR: 5 [3; 7]. During hospital stay, four patients died (in-hospital mortality: 2.1%), and nine others died within 30 days of admission (30-day mortality 4.8%).

A multivariable analysis was conducted to identify factors influencing 30-day mortality, in-hospital mortality and length of hospitalisation, focusing on gender, age, ISS and FISS. The analysis revealed a significant association between higher ISS and increased in-hospital mortality (Odds Ratio (OR) 0.64, 95% Confidence Interval (CI) 0.26; 1.60, *p*=0.342]), 30–day mortality (OR 1.06, 95% CI 0.82; 1.37, *P*<0.639), except for hospital stay (OR 0.57, 95% CI 0.35; 0.80, *p*<0.001).

The severity of facial trauma was evaluated using a modification of the Facial Injury Severity Scale (FISS), which ranges from 1 (least severe) to >9 (most severe), whereby lacerations were included regardless of their length. Most patients experienced moderate facial trauma, typically scoring around 2 on the FISS (IQR 2, [1; 3]. Only a small proportion of patients endured severe facial trauma. These results indicate that the majority of maxillofacial trauma cases fall within the least and moderate severity range Table 5.

**Table 5:** Multivariable analysis to determine the contributing factors (gender, age, FISS) for hospital stay, 30-day mortality and in-hospital mortality. A total of 188 observations were included in the final model; AUROC (30-days mortality) = 0.703; AUROC (in-hospital mortality) = 0.794

**Multivariable analysis**	**Coef. / Odds ratio**	**95% CI**	***p*-value**
**Duration of hospitalisation [days]**			
Gender	1.11	(-0.11; 2.32)	0.073
Age	0.02	(-0.05; 0.10)	0.519
FISS	0.57	(0.35; 0.80)	<0.001
APT	0.00		
AC	1.17	(-0.15; 2.49)	0.082
**In-hospital mortality**			
Gender	0.81	(0.10; 6.18)	0.702
Age	1.14	(0.96; 1.35)	0.103
FISS	0.64	(0.26; 1.60)	0.342
APT	1.00		
AC	0.96	(0.09; 9.94)	0.974
**30-days mortality**			
Gender	1.54	(0.36; 6.52)	0.556
Age	1.15	(0.98; 1.20)	0.119
FISS	1.06	(0.82; 1.37)	0.639
APT	1.00		
AC	1.57	(0.48; 6.69)	0.540

### Total costs

The average hospitalisation cost for all 188 patients was 1162.30 CHF, IQR [487.14; 1979.35] per patient. No statistically significant differences were found in the median cost per patient between genders, with women incurring 1218.64 CHF, IQR [540.20502; 2032] and men 1026.14 CHF, IQR [437.94501; 1958.5601], *p*=0.571. Additionally, the cost differences were not significant when comparing patients on AC or APT (*p*=0.084), as shown in Table 6.

**Table 6:** Multivariable analysis to determine the contributing factors (gender, age, FISS) for total costs. A total of 188 observations were included in the final model

**Multivariable analysis**	**Coef. / OR**	**95% CI**	***p*-value**
**Total Costs (CHF)**			
Gender	376.17	(-1166.47; 1918.81)	0.631
Age	49.69	(-47.10; 143.33)	0.320
FISS	-50.54	(-342.74; 241.65)	0.733
APT	0.00		
AC	536.24	(-1144.08; 2216.56)	0.530

**Fig. 5:**
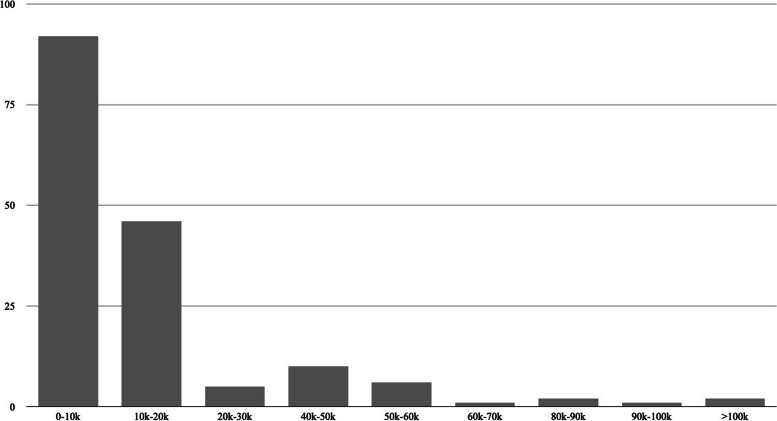
Total care costs per patient are broken down in increments of 10,000

Costs up to 10,000 CHF were incurred by 48.9% of the patients (*n*=92), with total costs per patient illustrated in Fig. 5.

## Discussion

In recent years, there has been a rising trend in maxillofacial injuries among the elderly population [27, 28].Although maxillofacial trauma is governed by the same fundamental principles regardless of age, when assessing older trauma patients, it should be taken into account that trauma has a greater physical impact on the older age group, due to their decreased physical reserves and age-related coexisting conditions including cardiovascular disease, poor eyesight, osteoporosis, atrophy of muscle mass, arthritis, and cognitive decline [28, 29]. Specific organ system dysfunctions (such as ischemic heart disease and dysrhythmias) and polypharmacy may also contribute [30]. In addition, given the widespread use of anticoagulation and antithrombotic therapy in this age group, understanding strategies of antithrombotic management in these patients is of key importance [31]. Therefore, this retrospective study aimed to assess the epidemiology, mechanisms, complications, treatment, and outcome of maxillofacial trauma in geriatric patients receiving AC/APT.

A total of 188 maxillofacial injuries in senior patients (>65 years) receiving AC/APT were recorded from 2013 to 2019. The mean age of the total study population was 81 years. Fifty-four percent of all patients were over 80 years of age. In contrast to other studies where the number of females with maxillofacial trauma was significantly higher compared to men [28], the proportion of male inhabitants was higher in our study, probably representing the higher incidence of cardiovascular and atherothrombotic diseases in men and thus the indication for AC/APT [32, 33] . However, in line with other studies highlighting the higher life expectancy of women, our study revealed that female patients had a significantly higher age than men.

As previously mentioned, comorbidities likely constitute one of the foremost factors to be considered in managing patients with maxillofacial trauma. As expected in our patients, cardiovascular diseases and atrial fibrillation were the most common pre-existing medical entities, whereas associated metabolic diseases, psychiatric, and neurological disorders were also observed. The high incidence of cardiovascular comorbidities in our study population may also explain the high occurrence of syncope in our patients. Indeed, in geriatric patients, trauma resulting from falls is more likely to be associated with conditions like syncope, acute seizures, stroke, myocardial infarction, transient ischemic attacks, and other acute medical issues. The latest is important when assessing a geriatric injury resulting from a fall followed by a loss of consciousness because serious underlying systemic disorders like severe cardiac arrhythmias or thromboembolic diseases could potentially be masked. Hence, healthcare professionals should remain vigilant for any signs or symptoms of neurological and cardiovascular diseases that might necessitate further evaluation [34].

In agreement with prior research emphasising falls as the primary cause of facial fractures in elderly patients [4, 21, 31, 34] , we observed that falls accounted for the majority of facial trauma in our study population, with 157 cases (83.5%). However, we observed no differences between genders in the impact of falls. This is in contrast to previous studies demonstrating a higher proportion of falls in female individuals due to the menopausal redistribution of body mass index and later retirement status [35, 36].

In view of the aging world population and the increased risk of falls among older people, an increasing number of falls can be expected in the future [37]. Moreover, falls are linked to substantial health repercussions, including bone fractures, hospital admissions, and institutionalisation [38–40] , thus resulting in substantial related costs. Indeed, in a prospective study by Woolcott et al. (2012), the average cost of a fall resulting in an ED visit was estimated to be $11,408 [41] . If hospitalisation was required, the average cost of a fall increased to $29,363. Our results indicate that the average cost of emergency department care for patients with facial fractures on AC/APT is approximately 10,000 CHF. Median hospital costs of all 188 patients amounted to 1,162 CHF per patient (IQR 487 – 1,979 CHF).

The second most frequent cause of facial trauma in our study was bicycle accidents, and this is consistent with earlier studies [39]. Indeed, bicycle-related accidents often lead to head and facial injuries, with facial injuries observed in 34% of cyclists admitted to the ED for their trauma [42]. In our study, bike accidents were significantly more frequent in men than in women. Typically, male drivers have a tendency to downplay the impact of aging, which encompasses decreased reaction times and impaired audiovisual abilities, factors that can heighten their susceptibility to traffic accidents [43]. It should be mentioned, however, that the findings of the studies are controversial, with some supporting an equivalent impact of maxillofacial trauma after bike accidents by gender [44]. Nevertheless, the data regarding the geriatric population are scarce.Our study shows similar findings to other studies regarding the location of facial fractures, with a higher incidence of orbital and cheekbone fractures, followed by nasal bone fractures. Given that our facility operates as a Level 1 trauma centre, there is a significant aggregation of facial trauma cases [45]. Furthermore, it is atypical for elderly patients with such injuries to seek treatment at private hospitals or clinics, largely because their associated diseases and comorbidities necessitate management in an acute hospital setting. Additionally, the availability of maxillofacial surgeons in other hospitals is significantly reduced. Since we have observed that a quarter of the patients on anticoagulants or antiplatelet therapy (AC/APT) experience brain haemorrhages, neurosurgical assessments are necessary, which are practically available only in major hospitals. This could explain how the large number of 71 patients (37.8%) referred from other hospitals in this study came about. Another possible explanation is the high impact of syncope in our study population (17.0%, *n*=32). Indeed, patients with loss of consciousness tend to sustain more severe maxillofacial injuries after simple falls than those without loss of consciousness [46, 47].

Regarding bleeding complications, out of all 188 patients who were examined, 129 (68.6%) had a haematoma of the face, with the middle third of the face being most frequently affected (63.8%, *n*=120). More than a quarter suffer from cerebral haemorrhages (28.2%, *n*=53), with women (40.8%, *n*=20) being significantly more affected than men (23.7%, *n*=33). Twenty-three patients had epistaxis, and ten patients suffered from oral bleeding. Women (76.2%, *n*=64) were significantly more likely (*p*=0.044) to have a facial haematoma than men (62.5%, *n*=65), with haematomas of the lower third of the face, in particular, being significantly more common in women (9.5%, *n*=8) than in men (2.9%, *n*=3). One explanation for this could be the higher median age of the women in this study (IQR 84 [75.5; 87]) compared to the men (IQR 79.5 [74; 86]) and thus the higher prevalence of dermatoporosis of the women compared to the men [47].

Coexisting secondary trauma-related diagnoses consisted of fracture of the extremities in a substantial proportion of patients, chest trauma, and abdominal trauma in a limited minority of patients. Bleeding complications included intracerebral haemorrhage, retrobulbar/intraorbital haematoma (RHB), epistaxis, oral bleeding and others (one case of haemorrhage from the iliac artery). Epidemiological data on i.e. the occurrence of traumatic retrobulbar hematomas, epistaxis or oral bleeding is limited. The incidence of traumatic RHB is difficult to quantify with 3.2% after orbital surgery [48]. In a 15-year Swiss observational study, 26 patients were diagnosed with RHB following trauma among other things. Falls were the most common cause, with more than half of the patients being over 60 years old [49]. These findings are similar to our results.

The median length of hospitalisation was five days. There was no significant difference in in-hospital and 30-day mortality in relation to age and gender. Even though studies comparing mortality in patients with facial trauma with respect to the effects of anticoagulants and antiplatelet drugs are rare, in-hospital mortality in our study was comparable to the mortality of nonanticoagulated geriatric patients with traumatic brain injury after low-level falls [4, 50]. This aligns with the findings of Moyer et al., who demonstrated no notable difference in in-house mortality when comparing trauma patients with and without AC/APT prior to trauma [51]. Moreover, in a recent, large multicentre study, the use of pre-injury anticoagulation and antithrombotic agents in elderly individuals with traumatic brain injury after ground-level falls showed no significant rise in mortality, suggesting that the utilisation of AC/APT may have minimal influence on the clinical management of these patients [52, 53]. In the multivariate analysis, FISS was found to be significant determinant of length of hospitalisation, which correlates with previous study results [22].

Despite the strengths of the present study, some limitations should be considered. Documentation bias cannot be entirely excluded in any retrospective study, despite carefully reviewing all included data. As this is a retrospective study, missing data cannot be avoided entirely, despite the efforts made to ensure completeness of data extraction and to minimise the number of missing values. However, these biases can be expected to be equally distributed across all patient groups and are, therefore, unlikely to affect the study's conclusions. Further prospective multicentre studies in collaboration between the ED and Cranio-Maxillofacial Surgery are recommended. Therefore, a larger number of patients under the influence of anticoagulants and antithrombotic agents should be included for a detailed analysis of the effect of medication on complications after facial trauma in older people. Furthermore, our study did not incorporate long-term follow-up data that could provide additional insights into health costs and the persistence of disability.

## Conclusion

This study is the first to specifically investigate the epidemiology of maxillofacial trauma in geriatric patients receiving anticoagulant or antiplatelet therapy (AC/APT) prior to injury. Falls are the main cause, with a clear correlation between male gender and bicycle accidents. The most common diagnoses include orbital, zygomatic, and nasal fractures. Haemorrhagic complications were predominantly facial hematomas, especially in the middle third of the face, with intracranial bleeding being the second most common. Similar to retrobulbar hematomas, these complications were significantly more frequent in women. Surgical interventions for haemorrhagic complications were required in 8% specifically in the maxillofacial area. The in-hospital mortality rate was comparable to that of non-anticoagulated geriatric patients with traumatic brain injury due to low-severity falls, highlighting the need for further investigation into the effects of specific anticoagulation and antiplatelet agents in this population.

## Data Availability

The data underlying this article will be shared on reasonable request to the corresponding author.

## Abbreviations

AC: Anticoagulation
APT: Antiplatelet therapy
ASS: Acetylsalicylic acid
CI: Confidence interval
CHF: Confoederatio Helvetica Franc
DOAC: Direct oral anticoagulant
ED: Emergency department
FISS: Facial Injury Severity Score
ISS: Injury Severity Score
IQR: Interquartile range
LMWH: Low-Molecular-Weight Heparin
OD: Odds Ratios
P2Y12Ι: P2Y12Ι receptor Inhibitors
USA: United States of America
VKA: Vitamin K Antagonist
VTE: Venous Thromboembolism
